# Lung disease as a determinant of cognitive decline and dementia

**DOI:** 10.1186/s13195-015-0116-3

**Published:** 2015-03-21

**Authors:** James W Dodd

**Affiliations:** Academic Respiratory Unit, School of Clinical Sciences, University of Bristol, Learning & Research Building, Southmead Hospital, Bristol, BS10 5NB, UK

## Abstract

Almost 40 million people currently live with dementia but this is estimated to double over the next 20 years; despite this, research identifying modifiable risk factors is scarce. There is increasing evidence that cognitive impairment is more frequent in those with chronic lung disease than those without. Chronic obstructive pulmonary disease affects 210 million people, with cognitive impairment present in 60% of certain populations. Co-morbid cognitive dysfunction also appears to impact on important outcomes such as quality of life, hospitalisation and survival. This review summarises the evidence of an association between cognition, impaired lung function and obstructive lung disease. It goes on to examine the contribution of neuro-imaging to our understanding of the underlying pathophysiology. While the mechanisms of brain pathology and cognitive impairment are likely to be complex and multi-factorial, there is evidence to suggest a key role for occult cerebrovascular damage independent of traditional vascular risk factors, including smoking.

**Electronic supplementary material:**

The online version of this article (doi:10.1186/s13195-015-0116-3) contains supplementary material, which is available to authorized users.

## Introduction

The World Health Organization reports that 35.6 million people currently live with dementia but this is estimated to double over the next 20 years; despite this, research identifying modifiable risk factors is scarce. Mild cognitive impairment (MCI) is associated with a 5 to 10% annual conversion rate to dementia [1,2]. However, MCI is considered a potentially reversible state and not all of those with MCI go on to develop dementia. Therefore, clarifying which features predict progression to dementia and identifying modifiable targets is currently of great interest. The diagnosis of MCI generally requires the exclusion of co-morbid illness but there have been concerns about the generalisability of this approach given that 50% of those with MCI are thought to have a medical co-morbidity [3]. Chronic lung disease is one such medical co-morbidity with increasing evidence of an association with cognitive dysfunction and brain pathology.

## Lung function and cognitive impairment

**Figure 1:**
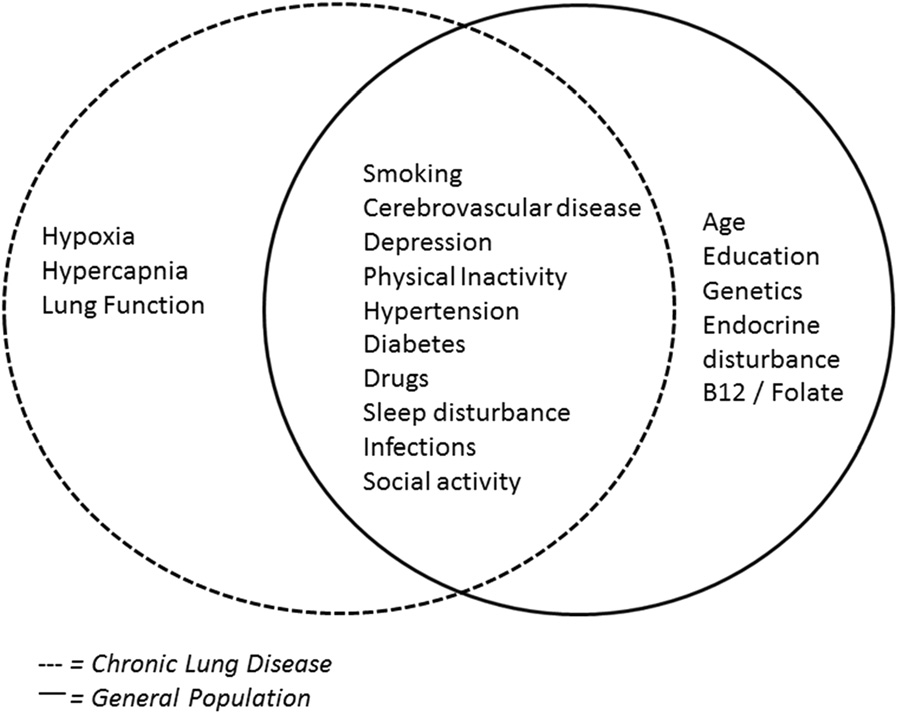
**Risk factors for cognitive impairment in both general and chronic lung disease populations.** This material has not been reviewed by the European Respiratory Society prior to release; therefore, the European Respiratory Society may not be responsible for any errors, omissions or inaccuracies, or for any consequences arising therefrom, in the content. Reproduced with permission of the European Respiratory Society [4].

Individuals with chronic lung disease are thought to be at an increased risk of cognitive decline. This may be as a result of risk factors which occur more frequently in those with lung disease (that are already known to negatively impact on cognition, such as smoking and hypertension) and/or as a direct result of respiratory limitations (such as hypoxaemia). Figure 1 summarises some of the overlapping risk factors for cognitive impairment in both general and chronic lung disease populations. However, importantly it seems that there is an association between cognitive impairment and lung disease independent of these factors [4].

Lung function is most often measured by spirometry, giving forced expiratory volume in 1 second (FEV1) and forced vital capacity, standardised for age and gender. The association between lung function and cognition has been tested in a number of large healthy population studies, particularly in elderly groups [4]. The majority of studies support at least some independent association between lung function and cognitive performance (Table 1).

**Table 1:** **Lung function and cognition**

**Study design**	**Population**	**Comment**	**Reference**
Longitudinal cohort 1990-2005	N = 10,975 men + women aged 47–70 years. Atherosclerosis Risk in Communities (ARIC) Study	Reduced lung function was associated with worse performance in cognitive assessments and with an increased risk of dementia hospitalization. No association was found between lung function and cognitive decline over time	[5]
Vietnam veteran study	N = 4,256 men aged 20 years	Poor cognitive ability in mid-life associated with reduced lung function in mid-life. Effects size small. Similar effects in non-smokers	[9]
Prospective observational study	N = 864 normative aging study. 12 year follow-up	Baseline FEV1 associated with visuo-spatial and global ability. Higher FEV1 associated with slower decline only in attention. Rate of decline in FEV1 not associated with cognition. Overall limited evidence of a relationship between FEV1 and cognitive decline	[6]
The Age, Gene/Environment Susceptibility Reykjavik Study: 23 year follow-up	N = 3,635; N = 1,281 subset 2/3 serial FEV1 over 7.8 years	Low mid-life FEV1/height predicted poor memory, processing speed, executive function MCI and dementia 23 years later. Decline in lung function over 7.8 years in mid-life was not associated with MCI or dementia	[8]
Longitudinal population Swedish Twin Study	N = 832 (50–85 year olds); 19 year follow-up	Changes in lung function led to subsequent changes in psychomotor speed and spatial abilities. No evidence that declines in cognitive function lead to subsequent decline in lung function	[83]
Longitudinal 23 year follow-up	N = 3,036 Japanese American males in Hawaii	Baseline FEV1 predict cognitive function (Cognitive Abilities Screening Instrument - CASI)	[84]
Healthy Longitudinal All ages	N = 2,551	FEV1 associated with cognitive function in all age groups, although significant associations were weak	[10]
MRC National Survey of Health and Development	N = 1,778 men and women	Mid-life FEV1 associated with mid-life psychomotor speed and decline over 10 years	[7]

FEV1, forced expiratory volume in 1 second; MCI, mild cognitive impairment; MRC, Medical Research Council.

The most comprehensive study comes from a longitudinal analysis of over 10,000 healthy men and women with repeated cognitive assessments between 1990 and 2006. This showed that impaired lung function was independently associated with worse cognitive function at baseline and higher subsequent risk of dementia hospitalization. However, no association was found between lung function and cognitive decline over time [5]. Other studies have shown mixed results, and where significant associations have been found they are not universal [6]. For example, mid-life lung function predicts mid-life psychomotor ability, memory, processing speed and executive function, but only psychomotor ability declined significantly over time [7,8]. Age is understood to be the most significant predictor of cognition and FEV1 also declines with age. However, FEV1 has been shown to be significantly and independently associated with cognitive function in all ages groups, though correlations can be weak [9,10].

Overall it appears that lung function is independently associated with measures of cognition and rate of cognitive decline. A lack of standardised neuropsychological assessments and variability in adjustment for confounders between studies currently limit clinical interpretation.

## Obstructive lung diseases

### Chronic obstructive pulmonary disease

One of the most common causes of impaired lung function is chronic obstructive pulmonary disease (COPD), a preventable and treatable disease characterised by persistent airflow limitation that is usually progressive and associated with an enhanced chronic inflammatory response in the airways and the lung to noxious particles or gases, most commonly tobacco smoke [11]. Unlike other common chronic diseases, the global prevalence of COPD has increased in recent years. In the UK, 10% of adults have an abnormally low FEV1 and 210 million people are diagnosed with COPD around the world. COPD is projected to become the third leading cause of death by 2030 [12], with smoking cessation the most effective intervention to reduce the risk of developing COPD and prevent disease progression [13,14]. Associations between COPD and cognitive function are explored in detail below.

### Asthma

It is estimated that 300 million people suffer with asthma globally, with prevalence rates of around 15.3% in England [15]. Asthma is defined clinically by the presence of symptoms (more than one of wheeze, breathlessness, chest tightness, cough) and of variable airflow obstruction. Unlike COPD, asthma predominantly affects children and young adults; it is most commonly associated with atopy and inflammation (allergy, eczema and hayfever) rather than smoking exposure.

Several studies suggest an association between neurocognitive dysfunction and asthma, citing mechanisms such as sleep disturbance, medication effects and systemic inflammation [16-26]. Mid-life asthma in particular has been associated with incidence of cognitive impairment and dementia (hazard ratios of 1.88 and 1.27, respectively), with risks increased further with exacerbation and hospitalisations [23-26]. In a study of 46 atopic patients with asthma, cognitive function was measured at baseline and 6 weeks after inhaled bronchodilators and steroids. This showed an improvement in cognition which appeared to relate to an improvement in variability of lung function, although it is not clear that practice and regression to the mean effects were fully accounted for [24]. A similar number of studies suggest no significant association between asthma and cognitive impairment [27-31]. The Childhood Asthma Management Program involved 1,041 children aged 5 to 12 years with asthma who were assessed prior to randomisation to receive anti-inflammatory medications. Neurocognitive performance was found to be normal and not associated with measures of asthma severity [27]. Flannery and colleagues [30] examined cognitive function in 11,578 children and found little support for a link between asthma and neurodevelopment problems. There were mixed results from an analysis of the Swedish Twin Registry, which showed a very modest longitudinal association between atopy and dementia (hazard ratio 1.16) in a large study of 22,188 people [20].

In many of these studies, sample sizes and multiple confounders in case definition limit definitive interpretation, leading some authors to conclude that socio-economic factors are mainly responsible for the variation in school performance and neurocognitive ability in asthma [32].

### Chronic obstructive pulmonary disease - a multi system disease

It is widely accepted that patients with COPD suffer with systemic manifestations beyond the lung and that these impact on disease management and further impair functional capacity, health-related quality of life and prognosis [33,34].

A UK study confirmed that COPD is associated with numerous co-morbidities; 2,699 patients with COPD in a UK General Practice research database were compared with age- and gender-matched controls. Amongst COPD patients, there was more frequent angina, cataracts, bone fractures and osteoporosis [35]. Importantly, these co-morbidities seem to be independent of smoking and traditional risk factors, suggesting a ‘COPD specific’ effect [36,37]. In addition it is thought that other frequently observed co-morbidities, including musculoskeletal weakness, diabetes, and depression, cannot be easily attributed to smoking [38].

The presence of these co-morbidities has a significant negative impact. In an analysis of over 20,000 subjects pooled from the Atherosclerosis Risk in Communities study and the Cardiovascular Health Study, an increasing number of co-morbidities was associated with a significantly increased risk of death at all stages of COPD severity. In addition, risk of hospitalisation within 5 years was increased in the presence of a number of co-morbidities including diabetes, hypertension and cardiovascular disease [39,40].

### Chronic obstructive pulmonary disease - co-morbid cognitive dysfunction and dementia

Estimates of cognitive dysfunction in COPD range from 10 to 61%, depending on the study population and method of neuropsychological assessment [4,41]. Cognitive impairment appears to be an important determinant of outcomes in COPD, with evidence that it is associated with poor quality of life, hospitalisation and reduced survival and is likely to profoundly influence an individual’s ability to manage their disease [4,42,43].

A 12 month retrospective database analysis of 126,106 US nursing home residents showed a concurrent diagnosis of COPD and dementia in 37.2%, and 62% of those with COPD also had short-term memory problems [44]. Moderate to severe cognitive impairment has been shown to be present in up to 61% of severely hypoxaemic individuals with COPD [41]. The majority of studies show that patients with COPD have either global impairment or deficits in attention, memory and learning and motor functions. In the combined Nocturnal Oxygen Therapy and the Intermittent Positive Pressure Breathing trials 42% of patients with COPD had moderate to severe cognitive impairment compared with 21% amongst the control group [45]. A large longitudinal study showed that self-reported diagnosis of severe COPD (defined by oxygen use or physical activity limitation) was associated with a more rapid decline in a questionnaire marker of cognitive performance over 6 years [46].

In a well conducted cross-sectional analysis, MCI was found in 36% of patients with moderate to severe COPD (versus 12% in controls) [47]. Two longitudinal studies quantify the risk of developing MCI in patients with COPD. The first found that a diagnosis of COPD in mid-life is independently associated with developing cognitive impairment in later life (hazard ratio 1.85) [23]. The other found that a diagnosis of COPD at baseline was associated with an 83% increased risk of non-amnesic MCI (hazard ratio 1.83 (95% confidence interval 1.04 to 3.23)) [48]. In addition there was a dose–response relationship between individuals with COPD duration of over 5 years at baseline and risk of MCI [48].

By contrast, two studies reported no significant cognitive impairment in COPD. In one, patients with mild hypoxaemia had worse verbal fluency compared with a control group, but not outside the normal range [49]. The other compared community-based COPD patients and a healthy group; no difference in Mini Mental State Examination (MMSE) was reported, although the COPD group may have also included cases of asthma - and MMSE does not measure executive or working memory functions [50].

In summary, COPD is consistently associated with an increase in the risk of cognitive impairment, cognitive decline and dementia. The severity and frequency appear more marked in those with more advanced disease.

## Mechanisms

Early studies of cognitive performance in COPD focused on hypoxaemia, but cognitive impairment is present in the absence of hypoxaemia and explains only a small proportion of the variance in cognitive ability in those patients with COPD who are hypoxaemic [4,42]. The acute neuropsychological effects of hypoxaemia have been studied in healthy volunteers; results suggest that it is responsible for minor deficits in complex reasoning, reaction times and word finding, in addition to reduced practice or ‘learning’ effect on repeated testing [51-53]. Little is known about the chronic effect of hypoxaemia in the absence of lung pathology. It has been suggested that oxygen-dependent enzymes important in the synthesis of neurotransmitters such as acetylcholine may be the pathophysiological pathway responsible for neuronal dysfunction during hypoxaemia [54].

Cognitive impairment is a known consequence of cerebral small-vessel disease and recent neuroimaging studies suggest that occult cerebrovascular damage plays a key role in brain damage and dysfunction in COPD [55,56]. Support for a vascular-mediated brain pathology is provided by a study in 202 individuals with dementia collected over 17 years. In this post-mortem study 45.5% had cerebral atherosclerosis, and in a subgroup of 52 who went on to have full autopsy, emphysema was present in 36.5% [57].

Arterial stiffness is a non-invasive measure of vascular function and accurately predicts cardiovascular and cerebrovascular events. Arterial stiffness is thought to contribute directly to end-organ vascular damage through reduced vessel compliance, excessive pressure pulsatility resulting in vascular remodelling and impaired blood flow [58-60]. There is evidence of increased aortic stiffness in COPD, independent of smoking, which also relates to degree of airflow limitation and percentage emphysema on thoracic computed tomography scan [60,61]. It has been suggested that arterial stiffness in COPD may be due to increased susceptibility to degradation of connective tissue or accelerated aging. These factors are also implicated in the development of emphysema, suggesting a potential shared pathophysiology between pulmonary and vascular disease in COPD [61].

A comprehensive review of vascular disease in COPD presents several plausible mechanisms, including systemic inflammation (interleukin-6, C-reactive protein), oxidative stress (through activation of matrix metalloproteinases), physiological stress (hypoxia, sympathetic nervous system activation), accelerated aging and protease/antiprotease imbalance. It is argued that many of these pathways are abnormal in COPD, independently predict cardiovascular disease and show direct pathophysiological links to the development of emphysema [62].

Acute exacerbations of COPD are events characterised by a change in baseline breathlessness, cough, and or sputum. Exacerbations are triggered by a combination of host and external factors, including airway infection and environmental pollution [63]. COPD exacerbations are associated with significant risk of death, with an in-hospital mortality of 7.7% and a 90-day mortality of 13.9% [64]. Cognitive function has been assessed among patients hospitalised with acute exacerbation and compared with individuals with COPD but without exacerbations and healthy controls [42]. In this study over half of those with exacerbation had moderate to severe cognitive impairment, most severely affecting executive function and associated with duration of hospitalisation and reduced quality of life. In the same study cognitive impairment did not appear to recover at 3 months. Whilst this could be due to pre-existing cognitive deficit, there are plausible mechanisms by which exacerbations may influence cognition and brain pathology. These relate to the acute physiological changes associated with infection and respiratory failure. In addition, recent work suggests that exacerbations are inflammatory episodes associated with arterial stiffness and myocardial injury [65].

## Brain pathology and lung disease

Smoking has been shown to be associated with a reduction in volume and density of frontal grey matter, risk of stroke, pre-clinical brain changes and cerebral atrophy on magnetic resonance imaging (MRI) [66,67]. Impaired lung function has been associated with greater cerebral white matter lesions, independent of conventional vascular risk factors, including smoking, in large population studies [68-70]. The Copenhagen Heart study showed a 30% increased risk of cerebral infarction amongst those with poor lung function (FEV1) [71]. In a smaller community sample, a significant association was observed between lung function and both cerebral atrophy and volume of white matter lesions in men with what was termed 'chronic respiratory disease', but not in women or healthy controls [72].

Table 2 summarises studies reporting on the relationship between brain pathology and COPD. Two recent case–control studies suggest that COPD is associated with reduced hippocampal and grey matter volumes, which appear to correlate with measures of disease severity and cognitive function [73,74]. A subgroup of patients on inhaler medication (that may have included individuals with COPD) from the Rotterdam population study had more severe periventricular white matter lesions than healthy participants [75]. However, a study on a smaller but well-defined sample of COPD patients with and without oxygen dependence found no difference between patients and healthy controls in either white matter lesion or cerebral tissue volumes [76]. More recently, a large population study showed that COPD was an independent risk factor for cerebral microbleeds at baseline and a significant increased risk of developing deep and paratentorial cerebral microbleeds over time, suggestive of hypertensive or arteriolosclerotic microangiopathy [55].

**Table 2:** **Neuroimaging and chronic obstructive pulmonary disease**

**Study design**	**Population**	**Cognitive measures**	**Imaging**	**Conclusions/comments**	**Reference**
Case control	6 severe COPD	NPT	MRI	SCOPD worse language, executive and visuo-spatial COPD smoked more and increased previous diabetes. No difference in brain volumes. SCOPD frontoparietal and periventricular white matter hyperintensities. Small differences? smoking related	[85]
	13 moderate COPD		T1		
	12 cognitive normal		Diffusion		
	? non-hypoxaemic				
Case control	25 stable COPD versus 25 controls	NPT	MRI	COPD worse MMSE, visual reproduction, figure memory test. COPD reduced GM volume. In COPD some regions correlated with oxygenation and regional GM volume was negatively correlated with disease duration. GM volume in inferior triangular frontal cortex in COPD correlated with picture memory score	[74]
			T1 and VBM		
Case control/longitudinal Rotterdam study	N = 165 COPD	-	MRI susceptibility weighted imaging	COPD greater CMBs at baseline independent of risk factors, including medications such as anti-thrombotics. COPD odds ratio 7.1 of developing deep/paratentorial CMB	[55]
	N = 645 controls				
Case control	N = 37 mild to moderate COPD	MMSE	MRI	The hippocampal volume was significantly smaller in COPD. It positively correlated with MMSE score, oxygen saturation in mild to moderate COPD patients, and levels of blood oxygen in both mild to moderate and severe COPD patients	[73]
	N = 31 controls				
Case control	25 stable non-hypoxaemic	NPT	MRI volumes, DTI, rfMRI	No age-related atrophy, reduced white matter integrity and increased resting state activation, whiter matter damage widespread and independent of traditional vascular risk measures, may account for cognitive impairment	[56]
	25 controls				
Case control	N = 25 stable	MMSE	MRI, DTI	Reduced GM density and increased fractional anisotropic values in COPD. Possible correlations with oxygen levels, visual tasks and disease duration. But small numbers, weak correlations	[78]
	N = 25 controls				
Case control	9 controls	NPT	MRI volumes	COPD had worse global cognition, memory, mood, but no difference in brain volumes or spectroscopy. Controlled for age gender and education	[76]
	18 COPD (9 oxygen-dependent)				
Cross-section non-demented elderly community cohort (N = 1,077)	Age 60–90 years	-	MRI	COPD diagnosis had more severe periventricular white matter lesions. But COPD and oxygen not associated with subcortical white matter lesions or lacunar infarcts. Low oxygen saturations independently associated with more severe periventricular white matter lesions	[75]
	Adjusted for age, sex, hypertension, DM, BMI, pack years, cholesterol, Hb, MI, LVH.				

BMI, body mass index; CMB, cerebral microbleed; COPD, chronic obstructive pulmonary disease; DM, diabetes mellitus; DTI, diffusion tensor imaging; GM, grey matter; Hb, haemoglobin; LVH, left ventricular hypertrophy; MI, myocardial infarction; MMSE, mini mental test examination; MRI, magnetic resonance imaging; NPT, neuropsychological test; rfMRI, resting-state functional MRI; SCOPD, severe chronic obstructive pulmonary disease; VBM, voxel-based morphometry.

Diffusion tensor imaging has been shown to be a more sensitive measure of white matter microstructural damage [77]. Recent small, cross-sectional case–control studies in COPD populations show widespread white matter microstructural damage independent of smoking [56,78]. In healthy older individuals, white matter microstructure in the corpus collosum correlated with cardiorespiratory fitness (oxygen uptake (VO_2_) peak r = −0.458) with subsequent tractography suggesting pre-frontal connections associated with motor planning [79].

Resting-state functional MRI measures low-frequency fluctuations in blood oxygen level-dependent signals in the brain at rest. In a study comparing stable non-hypoxaemic individuals with COPD to age-matched controls, patients with COPD had increased activation in seven out of eight of these networks. This increased activation of grey matter in COPD may reflect attempts to overcome damaged white matter pathways [56].

Finally, an MRI spectroscopy study in patients with non-hypoxic severe COPD showed that cerebral metabolism was significantly altered and that the pattern of derangement differed from that seen in heart failure and diabetes [80].

There are very few neuroimaging studies specifically in asthma. An MRI study of 17 people on long-term steroids suggests a possible reduction in hippocampal volume and declarative memory compared with controls [18]. In an ovalbumin-induced mouse model of asthma, there was evidence of impaired learning and hippocampal damage [81]. Finally, a recent MRI study of 19 to 47 year olds with mild to moderate asthma showed that incidental brain abnormalities were common (62%), of which 25% were white matter hyperintensities [82].

## Discussion and conclusions

The benefits of increasing our understanding of the relationship between chronic lung disease and cognitive impairment are two-fold. First, it may help to identify modifiable risk factors and therapeutic interventions to reduce the risk of developing dementia. Second, it provides the opportunity to reduce the impact of cognitive impairment on this vulnerable population with a chronic long-term condition, COPD.

Impaired lung function has been shown to independently predict cognitive performance but evidence of an association with cognitive decline is mixed. COPD is a common multi-system disease with growing evidence of an accelerated cognitive decline. The mechanisms of brain pathology and cognitive impairment are likely to be complex and multi-factorial but MRI suggests a potential COPD-specific cerebrovascular effect. This provides an appealing therapeutic target for reversing or halting cognitive decline in this population. Further studies are therefore required to clarify cerebrovascular mechanisms of brain pathology and cognitive impairment in COPD.

## Note

This article is part of a series on *The impact of acute and chronic medical disorders on accelerated cognitive decline*, edited by Carol Brayne and Daniel Davis. Other articles in this series can be found at http://alzres.com/series/medicaldisorders

## Abbreviations

COPD: chronic obstructive pulmonary disease
FEV1: forced expiratory volume in 1 second
MCI: mild cognitive impairment
MMSE: Mini Mental State Examination
MRI: magnetic resonance imaging
